# Dexmedetomidine attenuates lipopolysaccharide-induced acute liver injury in rats by inhibiting caveolin-1 downstream signaling pathway

**DOI:** 10.1042/BSR20204279

**Published:** 2021-03-04

**Authors:** Fei Tong, Wenchao Shen, Pengtao Song, Jiafeng Song, Yonghe Hu, Feifan Liu, Zhipeng Meng, Jing Liu

**Affiliations:** 1 Department of Anesthesiology, Huzhou Central Hospital, Affiliated Central Hospital HuZhou University, Huzhou, Zhejiang Province, China; 2 Department of Radiology, Huzhou Traditional Chinese Medicine Hospital Affiliated to Zhejiang Chinese Medical University, Huzhou, Zhejiang, China; 3 Department of Pathology, Huzhou Central Hospital, Affiliated Central Hospital HuZhou University, Huzhou, Zhejiang Province, China; 4 Department of Anesthesiology, Huzhou Maternal and Child Health Care Hospital, Huzhou, Zhejiang Province, China

**Keywords:** Dexmedetomidine, Acute liver injury, Lipopolysaccharide, TLR-4, NLRP3

## Abstract

Objective: The aim of the present study is to investigate the anti-injury and anti-inflammatory effects of dexmedetomidine (Dex) in acute liver injury induced by lipopolysaccharide (LPS) in Sprague–Dawley rats and its possible mechanism.

Methods: The acute liver injury model of male rats was established by injecting LPS into tail vein. The mean arterial pressure (MAP) of rats was recorded at 0–7 h, and lactic acid was detected at different time points. Wet/dry weight ratio (W/D) was calculated. Pathological changes of rat liver were observed by HE staining. ALT and AST levels in serum were detected. The activities of myeloperoxidase (MPO) and superoxide dismutase (SOD) in liver tissue homogenate and the levels of IL-1β and IL-18 in serum were detected by ELISA. Protein levels of Caveolin-1 (Cav-1), TLR-4 and NLRP3 in liver tissue were tested by immunohistochemistry method. The expression of Cav-1, TLR-4 and NLRP3 mRNA in liver tissue was detected by quantitative polymerase chain reaction (qPCR) to explore its related mechanism.

Results: Compared with NS group, serum lactic acid, W/D of liver tissue, MPO, SOD, IL-1β and IL-18 were significantly increased and MAP decreased significantly in LPS group and D+L group. However, compared with NS group, D group showed no significant difference in various indicators. Compared with LPS group, MPO, SOD, IL-1β and IL-18 were significantly decreased and MAP was significantly increased in D+L group. D+L group could significantly increase the level of Cav-1 protein and decrease the level of TLR-4 and NLRP3 protein in liver tissue caused by sepsis. The expression of Cav-1 mRNA was significantly up-regulated and the expression of TLR-4 and NLRP3 mRNA was inhibited in D+L group.

Conclusion: Dex pretreatment protects against LPS-induced actue liver injury via inhibiting the activation of the NLRP3 signaling pathway by up-regulating the expression of Cav-1 by sepsis.

## Background

Acute liver failure (ALF) is an extremely serious clinical syndrome caused by various factors. The clinical manifestations are liver tissue necrosis, cholestasis, liver fibrosis and function failure. There are numerous etiologies, including viral and autoimmune causes, but one of the common causes is endotoxemia-induced liver injury.

Dexmedetomidine (Dex) is a new generation of highly selective α2-adrenergic receptor agonists. It is also preferred by various clinical departments including anesthesia departments and intensive care units because of its analgesic, sedative, and antidepressant effects. Dexmetomidine can protect the brain, liver, stomach, heart and lung tissues by inhibiting apoptosis and anti-inflammation [1]. More and more studies have indicated that dexmetomidine may reduce the degree of acute liver injury caused by sepsis by inhibiting the release of pro-inflammatory cytokines mediated by TLR-4 signal pathway [2,3]. Most studies have shown that its downstream molecule is Caveolin-1 (Cav-1) protein, which may be involved in the regulation of TLR-4-mediated inflammation [4] and plays an important role in the occurrence and development of acute liver injury. Studies have shown that dexmetomidine can participate in the regulation of TLR-4-mediated inflammation through Cav-1 protein [5,6], which plays an important role in the occurrence and development of acute liver injury. TLR-4 also interacts with various signal-transduction pathways, such as NLRP3, NF-κB, TGF-β and other pathways. TLR-4 is an important signal pathway for the activation of NLRP3. Studies have also tackled NLRP3 inflammasome components, which can activate signaling pathway through inflammatory IL-1β and IL-18 release [7,8]. But the exact mechanism is not clear. In the present study, the effects of dexmedetomidine on inflammatory response, especially Cav-1, TLR-4 and NLRP3, were observed in rats with acute liver injury stimulated by lipopolysaccharide (LPS). This may be at least part of the mechanism that dexmetomidine inhibits TLR-4-mediated inflammatory signaling pathway. The present study will provide new insights into the mechanism of dexmedetomidine in reducing acute liver injury and provide a theoretical basis for clinical treatment of dexmetomidine.

## Materials and methods

### Materials

#### Animals

Forty adult male Sprague–Dawley rats, 7 weeks old weighing 200–250 g, were obtained from the Experimental Animal Center of Zhejiang Academy of Medical Sciences (license number: SCXK [Zhe] 2019-0002). The experiment was conducted in laboratory animal center of Zhejiang University. The experimental rats were housed in a comfortable and quiet room with a temperature of 22–25°C and a humidity of 40–60%. They were in a 12-h light–dark cycle (lights from 08:00 to 20:00), providing enough water and food. Laboratory Animal Management Committee of Zhejiang University approved the experimental protocol of the present study (Animal Ethics Review Batch No. ZJU20200046).

#### Reagents

The reagent dexmetomidine was purchased from Jiangsu Hengrui Pharmaceutical Co. Ltd. LPS assay kit was purchased from Sigma (U.S.A.). IL-1β ELISA kit and IL-18 ELISA kit were purchased from Neobioscience Co. Malondialdehyde (MDA) and superoxide dismutase (SOD) were purchased from Solarbio Life Sciences (China, Beijing). IL-1β and IL-18 ELISA kits were also purchased from Solarbio LifeSciences. Cav-1, TLR-4, NLRP3, GAPDH antibody were purchased from Abcam Biotechnology (Britain).

### Methods

#### Acute liver injury model induced by LPS

Before the beginning of the experiment, the rats were weighed and recorded, and then the rats were placed on a constant temperature heating pad. The rats were anesthetized by intraperitoneal injection of pentobarbital sodium in the supine position, and the blood pressure was monitored by puncture and catheterization of the common carotid artery. According to the guidance of previous articles, the model of acute liver injury was established by tail vein injection of LPS 8 mg/kg [9].

#### Surgical procedures

Rats were randomly divided into four groups, and each group comprised ten rats: after being anesthetized, within 0.5 h, all four groups were injected with the same volume of normal saline via tail vein. After 0.5 h, the dexmedetomidine (D) group and the dexmedetomidine and LPS (D+L) group, receiving intraperitoneal injection of Dex (50 μg/kg), the normal saline control group (NS) and the LPS group receiving tail-vein injection of equal volume 0.9% NS; 0.5 h later, LPS group and D+L group receiving tail-vein injection of LPS 8 mg/kg [10]; simultaneously NS group and D group receiving tail-vein injection of equal volume 0.9% NS. The experimental protocol details of every group are generalized in Table 1.

**Table 1:** Groups of animals and drugs administration

Groups	0 h	0. 5 h	1 h	3 h	5 h	7 h
NS	NS	NS	NS	NS	NS	Killed
LPS	NS	NS	LPS	NS	NS	Killed
D	NS	DEX	NS	NS	NS	Killed
D+L	NS	DEX	LPS	NS	NS	Killed

#### Measurement of mean arterial pressure and lactic acid

The right carotid artery of rats was catheterized for continuous arterial blood pressure monitoring. Samples of 0.5 ml of blood were drawn from the carotid artery at 0, 0.5, 1, 3, 5 and 7 h. Lactic acid (Lac) was measured by blood gas analyzer (Stat Profile PHOx, Nova Biomedical Corporation, U.S.A.). At the same timepoint, mean arterial pressure (MAP) [11] was recorded.

#### Measurement of the ratio of wet/dry weight ratio of liver tissue

After the rats were killed, the same parts and same mass of liver tissues were rinsed with phosphate buffer and then weighed with an electronic balance. After that, the liver tissues were roasted at 75°C in an oven for 72 h to weigh dry the liver tissues. The wet/dry weight ratio (W/D) of liver tissue was calculated.

#### Detection of liver tissue histopathology and immunohistochemistry

The left lobe of the liver was fixed with 10% formalin, embedded in paraffin, cut into 4-μm sections and stained with Hematoxylin–Eosin. Paraffin sections of liver tissue were incubated with primary antibody against Cav-1, TLR-4, NLRP3 at 4°C overnight, and then incubated with rabbit anti-goat polyclonal biotinylated antibody for 1 h at room temperature, and treated with horseradish peroxidase-labeled streptavidin at 37°C for 45 min. The sections were developed with diaminobenzidine and counterstained with Hematoxylin. The mean number of positive cells was evaluated from ten high power fields (×100) of each section in a double-blind manner. According to the DILI score system (pathological scoring system of DILl, DILI-PSS), the pathological degree of liver injury in each group was reflected (Table 2). DILl-PSS included hepatocyte steatosis 3 (vesicular 1, vesicular 2), hepatocyte cholestasis 1, apoptotic body 1, eosinophil infiltration 2, epithelial granuloma 1, and iron deposition in necrotic area 1, with a total of 9 points.

**Table 2:** Dili-pathological 5-point scale, DILI-Pathol 5-PS

DILI-PSS	DILI-Pathol 5-PS
8–9	Definite
7	Highly likely
5–6	Probable
3–4	Possible
1–2	Unlikely

#### Detection of liver function index in serum and concentration of inflammatory cytokines in liver tissue homogenate

Serum and plasma were collected after the rats were anesthetized and killed. The contents of ALT and AST in serum were measured by automatic biochemical analyzer. The liver tissue homogenate was prepared from ∼100 mg of liver tissue. The supernatant of the liver tissue homogenate was obtained after centrifugation, which was operated strictly according to the ELISA kit. The optical density was measured at 450 nm in spectrophotometer. The levels of IL-1β, IL-18, myeloperoxidase (MPO) and SOD in the samples were calculated based on a standard curve.

#### Detection of Cav-1, TLR-4 and NLRP3 mRNA by quantitative polymerase chain reaction

The total RNA of the cells was extracted and the reverse transcriptional expression was detected by SYBR Premix ExTaq II kit. The target genes Cav-1, TLR-4, NLRP3 and internal reference GAPDH primers (synthesized by Suzhou Jinweizhi Biotechnology Co., Ltd.) were all used. See Table 3 for details. The polymerase chain reaction (PCR) conditions were as follows: pre-incubation at 95°C for 10 min, followed by 40 cycles of denaturation at 95°C for 30 s, annealing at 55°C for 30 s and extension at 72°C for 20 s. Transcript levels of Cav-1, TLR-4 and NLRP3 mRNA expression in the liver tissues were quantitated by Δ-Δ threshold cycle (*C*_t_) method, using the housekeeping gene *GAPDH* for normalization. [11].

**Table 3:** Target gene Cav-1, TLR-4, NLRP3 and internal control GAPDH primer sequences

Gene	Primer sequences
Cav-1_Forward	5′-GCAGACGAGGTGAATGAGAAG-3′
Cav-1_Reverse	5′-GGTAGACAGCAAGCGGTAAAA-3′
TLR-4_Forward	5′-CAAGAAGCAACAACTTGACCTG-3′
TLR-4_Reverse	5′-CCTGTGAGGTCGTGAGGTIAG-3′
NLRP-3_Forward	5′-GCAGACGAGGTGAATGAGAAG-3′
NLRP-3_Reverse	5′-GGTAGACAGCAAGCGGTAAAA-3′
GAPDH_Forward	5′-TGACGTGCCGCCTGGAGAAA-3′
GAPDH_Reverse	5′-AGTGTAGCCCAAGATGCCCTTCAG-3′

### Statistical analysis

Statistical analyses were analyzed using SPSS 17. 0 software (SPSS; IBM Corporation, Armonk, NY). All data were expressed as the mean ± SD. The differences among the different groups were analyzed by one-way analysis of variance (ANOVA) and differences between the groups were evaluated using the *t* test for normally distributed data or the Mann-Whitney U test for abnormally distributed. *P*<0.05 was considered to be statistically significant.

## Results

### Comparison of MAP in four groups

**Figure 1:**
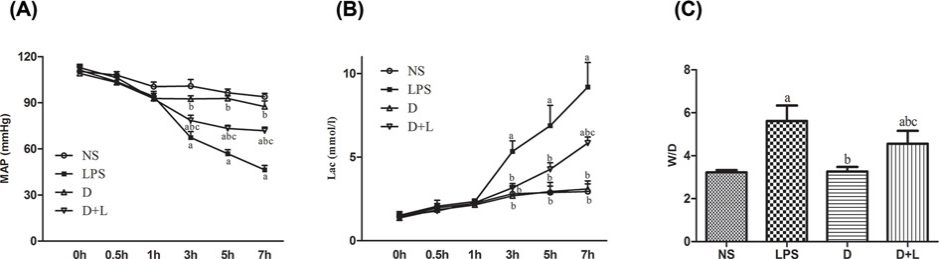
MAP, Lac and W/D weight ration in each group (**A**) The level of MAP in rats at different time points (*n*=10 samples/group). (**B**) The level of Lac in rats at different time points (*n*=10 samples/group). (**C**) The W/D weight ratio of liver in each group (*n*=10 samples/group). (a) Compared with NS group (*P*<0.05); (b) compared with LPS group (*P*<0.05); (c) compared with D group (*P*<0.05). There was no difference between NS group and D group.

Compared with the NS group, the MAP of the LPS and the D+L group decreased significantly at 3–7 h. Compared with the LPS group, the MAP of the D group and the D+L group increased significantly at 3–7 h. Compared with the D group, the MAP of the D+L group decreased significantly at 3–7 h (all, *P*<0.05) (Figure 1A).

### Comparison of Lac in arterial blood in four groups

Compared with the NS group, lac of the LPS group increased significantly at 3–7 h and the D+L group increased significantly at 7 h. Compared with the LPS group, the Lac of the D group and the D+L group decreased significantly at 3–7 h. Compared with the D group, the Lac of the D+L group increased significantly at 7 h (all, *P*<0. 05) (Figure 1B).

### Comparison of the W/D weight ratio in four groups

The ratio of wet weight to dry weight (W/D weight ratio) was used as an index of liver edema formation. Compared with the NS group, the W/D ratio of liver tissue in LPS group and D+L group which were injected with LPS increased significantly (*P*<0.05). However, compared with LPS group, pre-administration with Dex markedly decreased W/D weight ratio of liver tissue in D+L group rats (*P*<0.05). There was no difference between NS group and D group. This result was consistent with those of histological examinations (Figure 1C).

### Comparison of histopathological changes of liver injury and DILI-PSS in four groups

**Figure 2:**
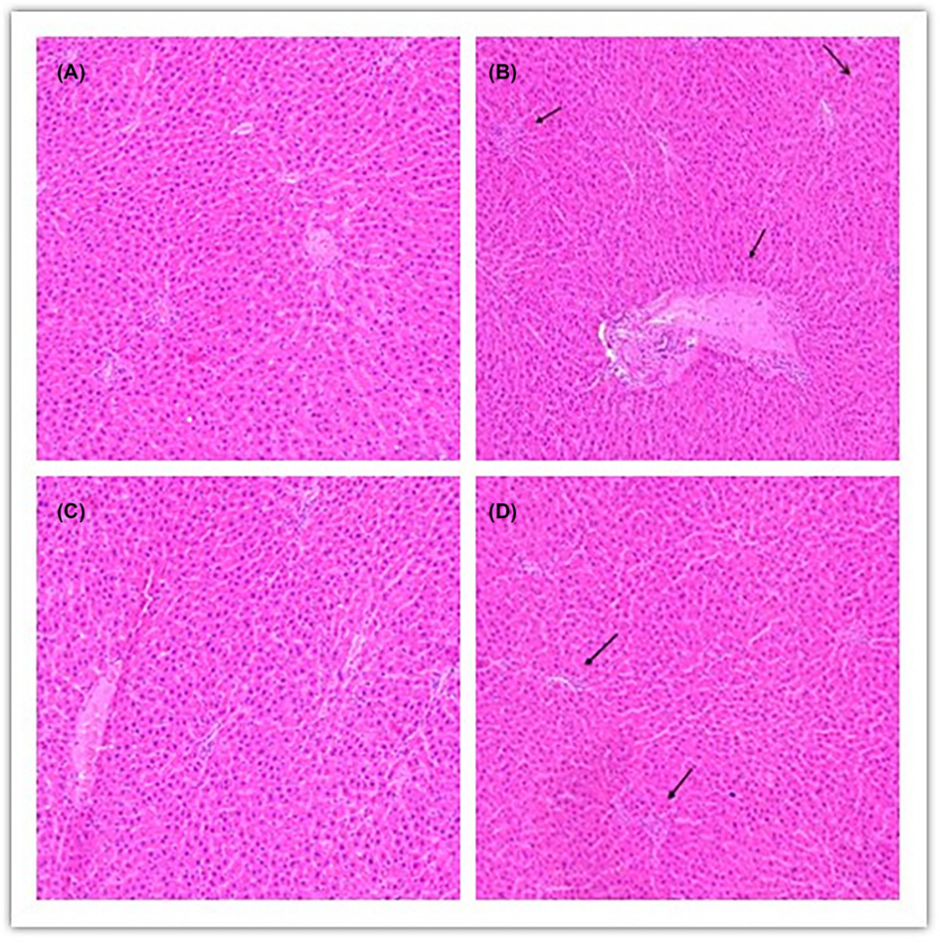
The histopathological changes of the livers in each group (HE staining, 100×) (*n*=10 samples/group) (**A**) NS group: the histological morphology of normal rat liver tissue, the structure of hepatic lobule is clear and complete, and the outline of liver is clear. The fine cell cords of the liver are arranged neatly, and the nucleus is located in the middle. No inflammatory cell infiltration and hepatocyte necrosis were found in the hilar area and around the central vein. (**B**) LPS group: the structure of hepatic lobule was damaged, the arrangement of hepatocytes was disordered, there was obvious inflammatory cell infiltration in the portal area and around the central vein, and the hepatocyte injury was serious. (**C**) D group: similar to NS group, there were no obvious pathological changes. (**D**) D+L group: the injury of liver tissue was significantly alleviated, the basement membrane around the portal vein was slightly thickened by congestion, the structure of hepatic lobule and hepatocyte cord was clear and intact, and the cell infiltration caused by inflammation was significantly improved.

**Figure 3:**
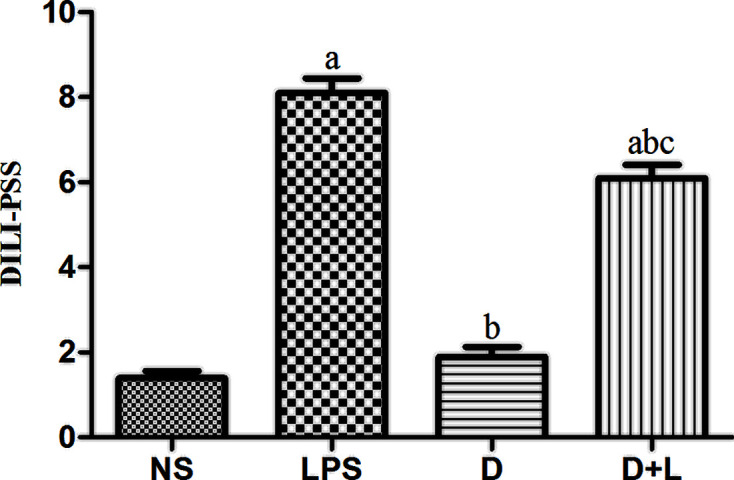
DILI-PSS in each group (*n*=10 samples/group) (**A**) Compared with NS group (*P*<0.05); (**B**) compared with LPS group (*P*<0.05); (**C**) compared with D group (*P*<0.05). There was no difference between NS group and D group.

In NS group, the structure of hepatic lobule was clear and complete, and the outline of liver was clear. The hepatocyte cords were arranged neatly, and the nucleus was located in the middle. No inflammatory cell infiltration and hepatocyte necrosis were found in the hepatic hilar area and around the central vein (Figure 2A). In LPS group, there was structural damage on the hepatic lobules, disordered arrangement of hepatic cells, obvious inflammatory cell infiltration around the portal area, central vein and severe damage of hepatocytes (Figure 2B). D group was similar to NS group, there was no obvious pathological change (Figure 2C). Compared with LPS group, there was some liver injury in D+L group. In D+L group, the injury of liver tissue was significantly improved, the structure of hepatic lobule and hepatocyte cord was clear and intact, and the fine cell infiltration caused by inflammation was significantly improved (Figure 2D). According to the DILI-PSS of four groups, the pathological diagnosis of NS group and D group was normal liver tissue. The pathological diagnosis of LPS group was acute hepatitis, which was caused by DILI definitely. The pathological diagnosis of D+L group was acute hepatitis, which was caused by DILI probably (Figure 3).

### Comparison of liver function indexes in serum in four groups

**Figure 4:**
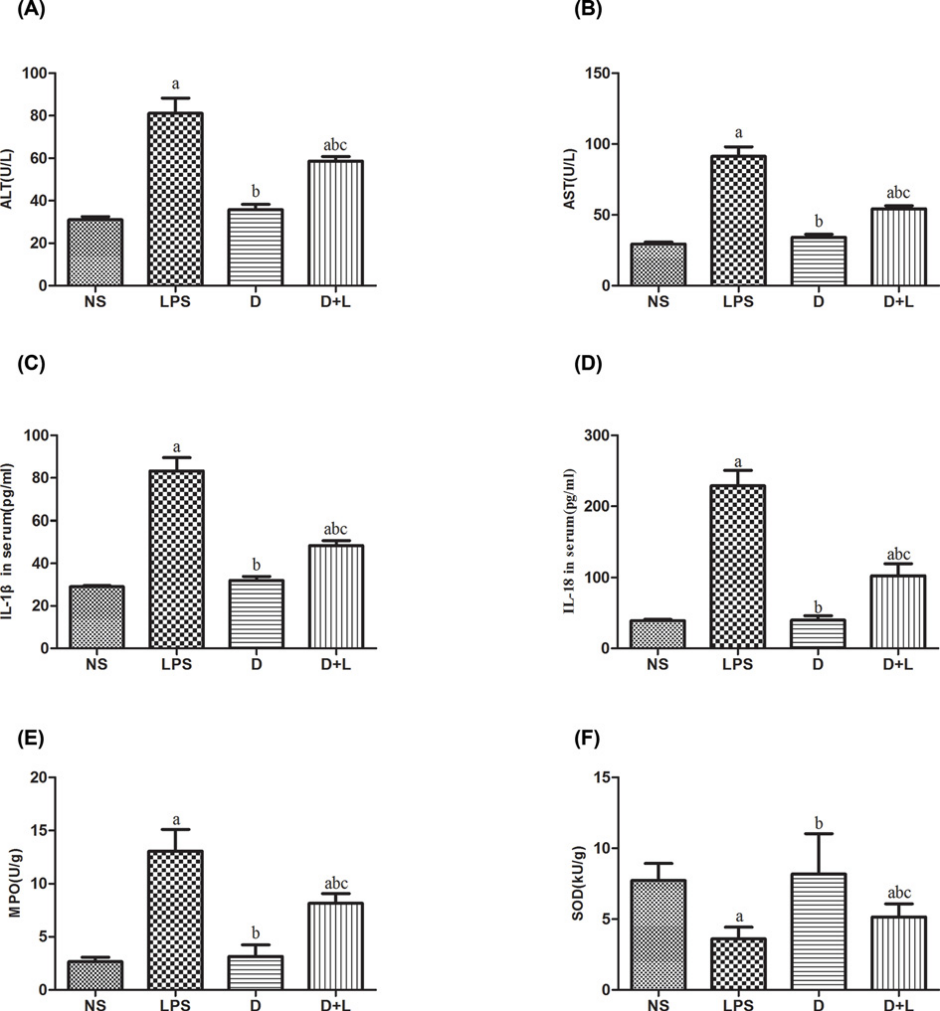
Liver function index and inflammatory factor index in each group (**A**) The level of ALT in each group (*n*=10 samples/group). (**B**) The level of AST in each group (*n*=10 samples/group). (**C**) The serum level of IL-1β in each group (*n*=10 samples/group). (**D**) The serum level of IL-18 in each group (*n*=10 samples/group). (**E**) The liver tissue level of MPO in each group (*n*=10 samples/group). (**F**) The liver tissue level of SOD in each group (*n*=10 samples/group). (a) Compared with NS group (*P*<0.05); (b) compared with LPS group (*P*<0.05); (c) compared with D group (*P*<0.05). There was no difference between NS group and D group.

The present study detected the effect of Dex on the liver function indicator. Compared with the NS group, the levels of ALT and AST, dramatically enhanced after LPS challenge in LPS group and D+L group (*P*<0.05). Dex pretreatment significantly reduced the above increase, and the concentration of actue liver injury related inflammatory cytokines in D+L group was lower than that in LPS group (*P*<0.05). There was no difference between NS group and D group. The results showed that dexmetomidine pretreatment could significantly inhibit LPS-induced ALT and AST (Figure 4A,B).

### Comparison of the levels of cytokines in liver tissue homogenate of four groups

In the present study, we further examined the effect of dexmedetomidine on the production of inflammatory cytokines. Compared with the NS group, the levels of IL-1β, IL-18 and MPO, dramatically enhanced after LPS challenge in LPS group and D+L group (*P*<0.05), while the level of SOD decreased significantly in LPS group and D+L group. The concentration of actue liver injury related inflammatory cytokines in D+L group was lower than that in LPS group (*P*<0.05), while the level of SOD in D+L group was higher than that in LPS group (*P*<0.05). There was no difference between NS group and D group. This result indicating that Dex pretreatment significantly inhibited LPS induced inflammatory cytokines (Figure 4C–F).

### The protein levels of Cav-1, TLR-4 and NLRP3

**Figure 5:**
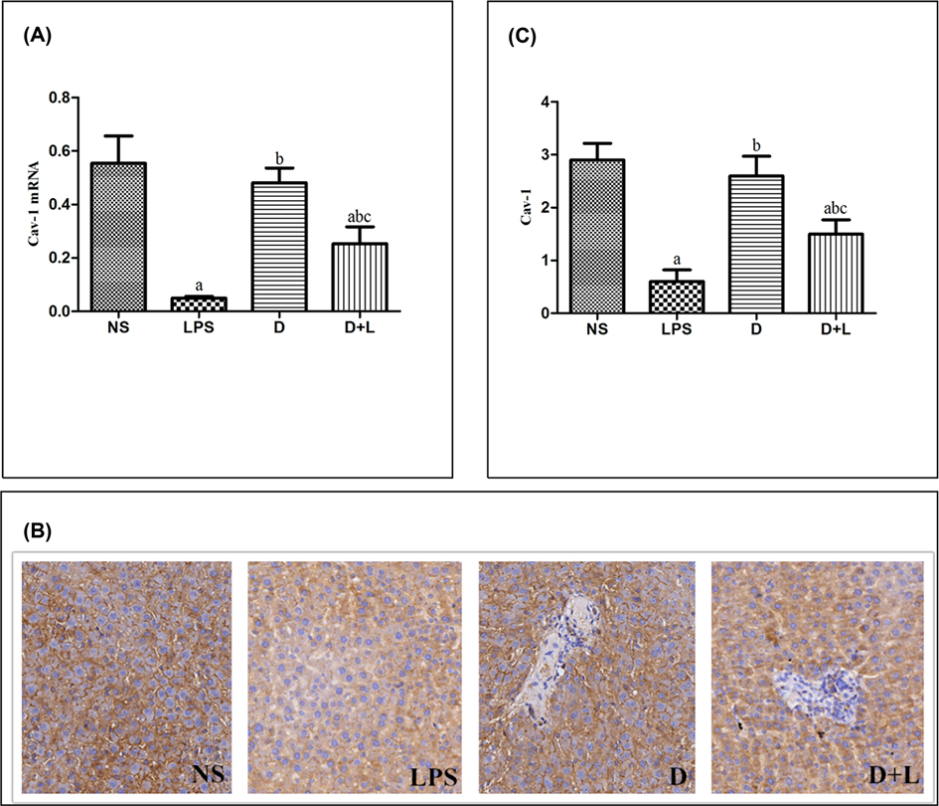
The level of Cav-1 mRNA, immunohistochemistry images and Cav-1 protein in each group (**A**) Quantitative reverse transcriptase-PCR for Cav-1 gene expression. Mean ± SD (*n*=10 samples/group). (a) Compared with NS group (*P*<0.05); (b) compared with LPS group (*P*<0.05); (c) Compared with D group (*P*<0.05). (**B**) Immunohistochemistry staining of Cav-1 (100×) (a) NS (b) LPS (c) D (d) D+L. (**C**) The protein level of Cav-1. Mean ± SD (*n*=10 samples/group). (a) Compared with NS group (*P*<0.05); (b) compared with LPS group (*P*<0.05); (c) compared with D group (*P*<0.05).

We measured the concentration of Cav-1 in liver tissue to evaluate whether dexmetomidine affected the production of Cav-1. In NS group and D group, the expression of Cav-1 protein was stable. The expression level of Cav-1 in LPS group and D+L group was significantly lower than that in NS group (*P*<0.05). Pre-treatment with Dex could significantly up-regulate the expression of Cav-1 which was markedly down-regulated by LPS. The expression level of Cav-1 in D+L group was significantly higher than that in LPS group (*P*<0.05). There was no significant difference between NS group and D group (Figure 5B and C).

**Figure 6:**
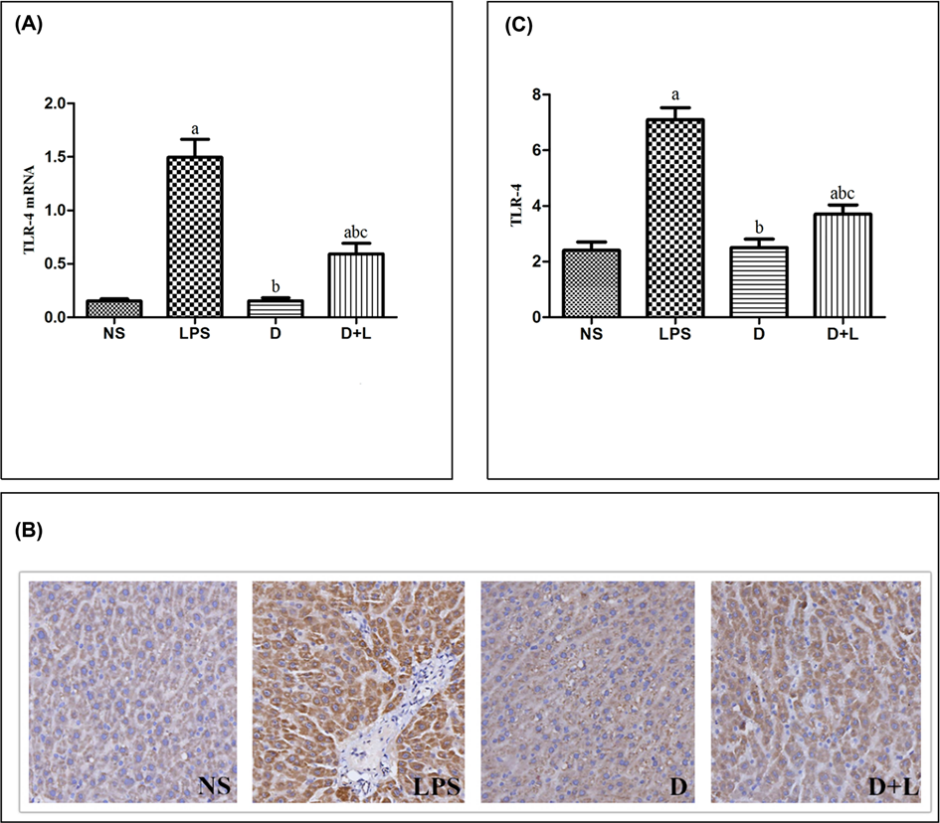
The level of TLR-4 mRNA, immunohistochemistry images and TLR-4 protein in each group (**A**) Quantitative reverse transcriptase-PCR for TLR-4 gene expression. Mean ± SD (*n*=10 samples/group). (a) Compared with NS group (*P*<0.05); (b) compared with LPS group (*P*<0.05); (c) compared with D group (*P*<0.05). (**B**) Immunohistochemistry staining of TLR-4 (100×) (a) NS (b) LPS (c) D (d) D+L. (**C**) The protein level of TLR-4. Mean ± SD (*n*=10 samples/group). (a) Compared with NS group (*P*<0.05); (b) compared with LPS group (*P*<0.05); (c) compared with D group (*P*<0.05).

In the NS group and D group, the expression levels of TLR-4 were stable. While the expression levels of TLR-4 in LPS group and D+L group were both dramatically higher than NS group (P<0.05). As pre-administration with Dex, the expression levels of TLR-4 in D+L group were both notably lower than LPS group (P<0.05). There was no significant difference between NS group and D group. This alteration indicated that TLR-4 up-regulation was related to LPS-induced actue liver injury, while Dex could protect against actue liver injury by decreasing the concentration of TLR-4 (Figure 6B and C).

**Figure 7:**
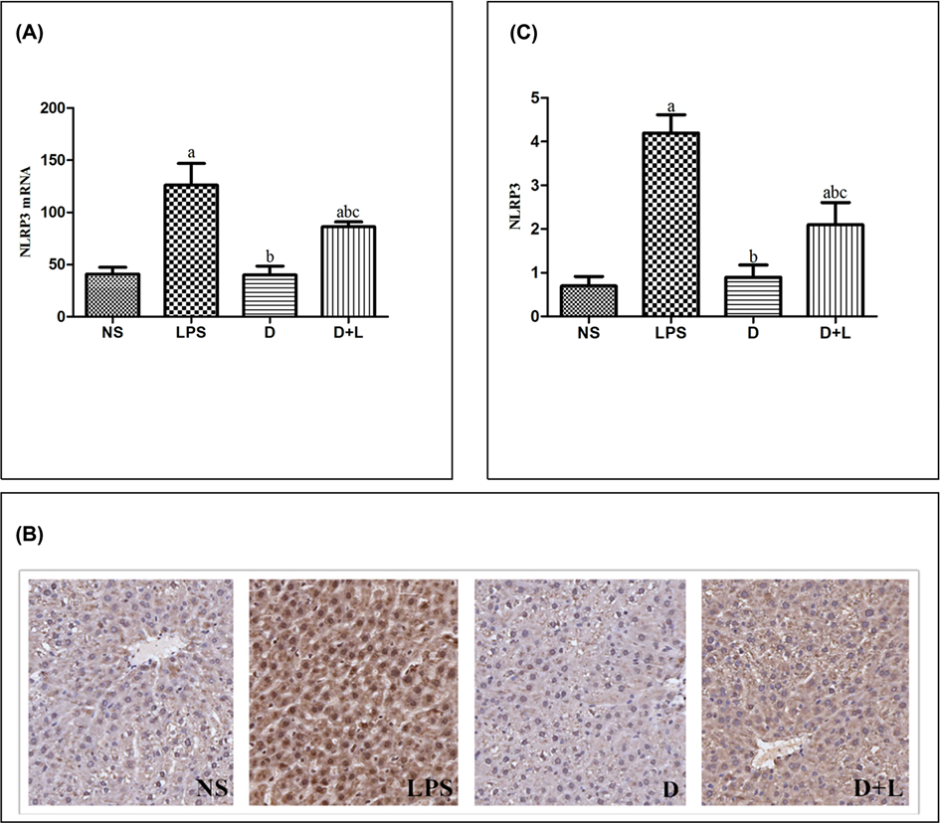
The level of NLRP3 mRNA, immunohistochemistry images and NLRP3 protein in each group (**A**) Quantitative reverse transcriptase-PCR for NLRP3 gene expression. Mean ± SD (*n*=10 samples/group). (a) Compared with NS group (*P*<0.05); (b) compared with LPS group (*P*<0.05); (c) compared with D group (*P*<0.05). (**B**) Immunohistochemistry staining of NLRP3 (100×) (a) NS (b) LPS (c) D (d) D+L. (**C**) The protein level of NLRP3. Mean ± SD (*n*=10 samples/group). (a) Compared with NS group (*P*<0.05); (b) compared with LPS group (*P*<0.05); (c) compared with D group (*P*<0.05).

NLRP3 signal pathway is an important part of the regulation of inflammatory response. In the NS group and D group, the expression levels of NLRP3 were stable. While the expression levels of NLRP3 in LPS group and D+L group were both dramatically higher than NS group (P<0.05). As pre-administration with Dex, the expression levels of NLRP3 in D+L group were both notably lower than LPS group (P<0.05). There was no significant difference between NS group and D group. This alteration indicated that NLRP3 up-regulation was related to LPS-induced actue liver injury, while Dex could protect against actue liver injury by decreasing the concentration of NLRP3 (Figure 7B and C).

### Quantitative PCR assay of Cav-1, TLR-4 and NLRP3 mRNA

In NS group and D group, the expression of Cav-1 mRNA was stable. The expression level of Cav-1 in LPS group and D+L group was significantly lower than that in NS group (*P*<0.05). Pre-treatment with Dex could significantly up-regulate the expression of Cav-1 which was markedly down-regulated by LPS. The expression level of Cav-1 mRNA in D+L group was significantly higher than that in LPS group (*P*<0.05). There was no significant difference between NS group and D group. This change was in accordance with the protein level of Cav-1 (Figure 5A).

The expression amounts of TLR-4 and NLRP3 mRNA were stable in the NS group and D group. While the expression amounts of TLR-4 and NLRP3 mRNA in LPS group and D+L group were both dramatically higher than NS group (*P*<0.05). As pre-administration with Dex, the expression levels of TLR-4 and NLRP3 mRNA in D+L group were both notably lower than LPS group (*P*<0.05). There was no significant difference between NS group and D group. These results was paralleled by that of the protein level of TLR-4 and NLRP3 and indicated that TLR-4 and NLRP3 mRNA up-regulation was associated with sepsis, while pre-treatment with Dex could relieve these changes (Figures 6A and 7A).

## Discussion

Cav-1 is an important structural protein of caveolae. Cav-1 is rarely found in normal liver tissues, but its expression changes in the state of liver disease. It is suggested that the changes of Cav-1 expression and function are related to the occurrence and development of liver disease [12,13]. Cav-1 plays an important role in the immune response against bacterial infection. Medina et al. [14] studied the effects of intravenous and oral administration of *Salmonella typhimurium* on mice. They found that Cav-1 has anti-infective effect [15,16], which can inhibit inflammatory reaction and reduce liver necrosis. Bacteria will produce a large amount of endotoxin after infecting the human body. Further studies have shown that Cav-1 has been located as a receptor for downstream molecules [17]. Cav-1 also regulates the signal chain of the central membrane of the cell, controlling several important signal chains. In further studies, Cav-1 has been shown to regulate the TLR pathway in hepatocytes.

TLR family is an important pathway related to inflammations [18–20]. TLR-4 is an important subpopulation of molecular clues of the family, which is expressed in endothelial cells, neutrophils and alveolar macrophages. It has been proved that TLR-4-dependent signaling pathway leads to the entry of early inflammatory cytokines into the liver, leading to acute liver injury, which is considered to be a fatal complication of septic shock [21]. The activation of TLR-4 signal pathway activates NLRP signal pathway, and NLRP signal pathway regulates immune response and inflammatory cytokines. Gurung et al. found that the activation of TLR-4 signaling pathway is an important mediator of acute liver injury during septic shock, and TLR-4 can activate NLRP3 through intermediate factors [22]. Recently, Murphy reported that the activation of TLR-4 receptor ultimately promotes the expression of NLRP3 and the assembly of inflammatory factors through the effect of CMPK2 on mitochondrial DNA [23].

The NLRs family in the cytoplasm is an important pathway related to inflammation. The NLRs family is divided into five subfamilies: NLRA, NLRB, NLRC, NLRP and NLRX. NLRP3 inflammatory complex is a response to stress signals, among which there are many studies on NLRP3 inflammatory complex. Importantly, more and more experimental and clinical studies have shown that the activation of NLRP3 pathway is an important mediator of acute liver injury during septic shock [24–26].

Dexmedetomidine is a highly selective α2-adrenergic receptor agonist, which has sedative, anti-anxiety, analgesic and hypnotic effects. In the experimental model, dexmetomidine can maintain perioperative hemodynamic stability by inhibiting the production and release of pro-inflammatory cytokines, thus protecting important organs such as brain and heart [27]. It has been reported that dexmetomidine can inhibit immune response by directly regulating the expression of Cav-1, thus reducing experimental acute liver injury.

Based on these studies, we speculate that dexmetomidine may regulate the NLRP3 inflammatory signal pathway by affecting Cav-1 protein [28]. The results showed that in terms of blood pressure fluctuation and lactic acid concentration, the blood pressure fluctuation and lactic acid concentration in the dexmetomidine group were significantly lower than those in the LPS group. To some extent, this reflects the therapeutic effect of dexmedetomidine on liver injury, but whether it is related to anti-inflammatory response is not clear and needs to be further studied [29,30].

In this experiment,we detected the expression of Cav-1, TLR-4 and NLRP3 in liver tissue of SD rats. The results showed that compared with NS group, LPS significantly decreased the expression of Cav-1 and significantly up-regulated the expression of TLR-4 and NLRP3. However, after the pre-injection of dexmedetomidine, compared with the LPS group, the expression of Cav-1 increased, while the expression of TLR-4 and NLRP3 decreased. It is suggested that dexmetomidine may play an anti-inflammatory effect in LPS-induced acute liver injury model by up-regulating Cav-1 and inhibiting the activation of NLRP3 pathway. In addition, studies have indirectly shown that only in LPS-induced inflammation, dexmetomidine can up-regulate the expression of Cav-1, thus inhibiting the activation of NLRP3 pathway. It is worth noting that the above indicators of D+L group did not reach the level of NS group or group D, indicating that the up-regulation of Cav-1 is not a single mechanism of dexmedetomidine against acute liver injury, and its complex molecular mechanism needs to be further studied.

There are certain limitations in the present study. The present study did not use the antagonist of the α2-AR. The role of α2-AR in mediating the beneficial effect of dexmetomidine on acute liver injury induced by sepsis is unclear.

## Conclusions

The present study indicates that pretreatment with dexmetomidine induced acute liver injury in rats by inhibiting the inflammatory pathways. The underlying mechanism is that dexmetomidine may up-regulate the expression of Cav-1 down-regulated by sepsis, which may conduce to the suppression of TLR-4/NLRP3-mediated signaling pathway, at least in part.

## Data Availability

All data (Figures 1–7) used to support the findings of the present study are available from Fei Tong (Contact e-mail: tongfei1990@foxmail.com) or the corresponding authors upon request.

## Abbreviations

Cav-1: Caveolin-1
Dex: dexmedetomidine
DILI: drug-induced liver injury
DILI-PSS: pathological scoring system of DILl
LPS: lipopolysaccharide
MAP: mean arterial pressure
MPO: myeloperoxidase
PCR: polymerase chain reaction
SOD: superoxide dismutase
W/D: wet/dry weight ratio
