# Correction: Dexmedetomidine attenuates lipopolysaccharide-induced acute liver injury in rats by inhibiting caveolin-1 downstream signaling pathway

**DOI:** 10.1042/BSR-2020-4279_COR

**Published:** 2021-06-02

**Keywords:** Acute liver injury, Dexmedetomidine, Lipopolysaccharide

This article is being corrected following notification from one of our readers alerting Bioscience Reports to duplicated sections within Figure 2 panels A-D. The Editorial Office contacted the authors and were provided with original images and raw data which did not contain the areas of concern raised by the reader. Following investigation by the Editorial Office, it was identified that the version of files accepted for publication on 8 February 2021 did not contain the image duplications that were identified post-publication. It has since been discovered that the duplications were introduced by an external production vendor during the production process prior to publication in order to achieve a coherent image style (i.e. modification of panel labelling within a figure), and thus first appeared in the Version of Record published by Bioscience Reports on 26 March 2021.

**Figure 2:**
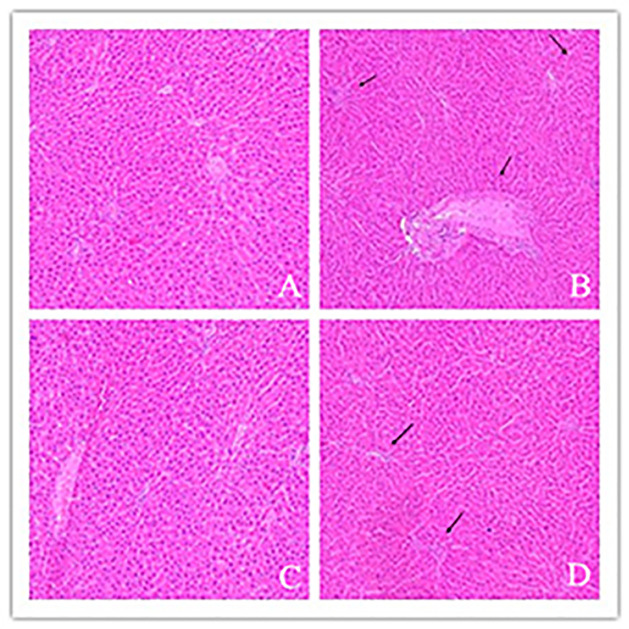
The histopathological changes of the livers in each group (HE staining, 100×) (*n* = 10 samples/group) (**A**) NS group: the histological morphology of normal rat liver tissue, the structure of hepatic lobule is clear and complete, and the outline of liver is clear. The fine cell cords of the liver are arranged neatly, and the nucleus is located in the middle. No inflammatory cell infiltration and hepatocyte necrosis were found in the hilar area and around the central vein. (**B**) LPS group: the structure of hepatic lobule was damaged, the arrangement of hepatocytes was disordered, there was obvious inflammatory cell infiltration in the portal area and around the central vein, and the hepatocyte injury was serious. (**C**) D group: similar to NS group, there were no obvious pathological changes. (**D**) D+L group: the injury of liver tissue was significantly alleviated, the basement membrane around the portal vein was slightly thickened by congestion, the structure of hepatic lobule and hepatocyte cord was clear and intact, and the cell infiltration caused by inflammation was significantly improved.

The correct version of Figure 2 as originally submitted by the authors and present in the accepted manuscript are included in this Correction. The Editorial Office apologises to both the authors of this paper and readers that such duplications were introduced. Immediate workflow changes have taken place within our production processes to prevent such errors occurring in the future.

